# Effects of a novel fulvic acid-enriched water-soluble fertilizer on potato growth, yield, and water-fertilizer use efficiency under different drip irrigation regimes

**DOI:** 10.3389/fpls.2025.1672560

**Published:** 2025-10-03

**Authors:** Fulin Xu, Yi Liu, Jiangtao Li, Jinquan Zhu, Nan Wu, Ajing Meng

**Affiliations:** ^1^ Institute of Agricultural Resources and Environment, Xinjiang Academy of Agricultural Sciences, Urumqi, China; ^2^ School of Hydraulic and Civil Engineering, Ludong University, Yantai, China; ^3^ Comprehensive Experimental Station, Xinjiang Academy of Agricultural Sciences, Urumqi, China; ^4^ National Soil Quality Akesu Observation and Experiment Station, Akesu, China; ^5^ School of Resources and Environmental Engineering, Ludong University, Yantai, China

**Keywords:** arid region, ‘Xisen 6’ variety, fertigation, water-fertilizer coupling, growth physiology, resource use efficiency

## Abstract

Northern China is a major potato-producing region, where water scarcity and low fertilizer use efficiency significantly constrain potato production. A field experiment was conducted in Xinjiang, China, in 2023 using the potato cultivar ‘Xisen 6’ to investigate the effects of a novel fulvic acid (FA)-enriched specialized fertilizer on potato growth, photosynthetic characteristics, yield, and water-fertilizer use efficiency under different drip irrigation levels. Three irrigation regimes were applied: conventional irrigation (W1: 4582.5 m^3^·ha^-1^), 15% deficit irrigation (W2: 3865.5 m^3^·ha^-1^), and 30% deficit irrigation (W3: 3165 m^3^·ha^-1^). Five fertilization treatments were implemented: no fertilizer (CK), conventional fertilizer (CF), and three FA-enriched formula fertilizers—F1 (FA: 120 g·L^-1^, N-P-K = 110-100-120), F2 (FA: 60 g·L^-1^, N-P-K = 130-120-150), and F3 (FA: 30 g·L^-1^, N-P-K = 170-150-130). The results indicated that both irrigation (W) and fertilization (F) significantly influenced potato growth, yield, and the efficiency of water and fertilizer use. Under W2 irrigation, the W2F2 treatment achieved optimal performance, with plant height, aboveground dry weight, SPAD, and tuber yield increasing by 56.67%, 71.24%, 21.69%, and 121.29%, respectively, compared to CK. WUE and PFP reached 13.84 kg·m^-3^ and 100.1 kg·kg^-1^, respectively, while maintaining a yield of 59.49 t·ha^-1^ under 15% water-saving conditions. Tuber yield exhibited highly significant positive correlations with plant height, aboveground dry weight, SPAD, net photosynthetic rate, WUE, and PFP. Principal component analysis based on growth, yield, and resource use efficiency identified W2F2 as the highest-ranked treatment. In conclusion, for ‘Xisen 6’ cultivation in Northwest China, applying formula fertilizer F2 (FA: 60 g·L^-1^, N-P-K = 130-120-150) under a drip irrigation rate of 3865.5 m^3^·ha^-1^ significantly enhances plant growth, yield, and water use efficiency, providing a sustainable strategy for optimizing potato production.

## 1 Introduction

Potato (*Solanum tuberosum* L.) is the world’s third most consumed and fourth most produced food crop, with an annual production of 376 million tons, ranking after wheat, rice, and maize. It is renowned for its high yield potential, stable productivity, and broad ecological adaptability, and is regarded as a vital crop in developing and least-developed countries, leading to its widespread cultivation globally (Aytekin and Çalışkan, 2024; Liu et al., 2025; Shrestha et al., 2024). Notably, China serves as a key region for global potato production, with its northwestern area being one of the primary production zones. However, limited water resources and low water-fertilizer use efficiency constrain sustainable agricultural development in this region (Wang et al., 2019). Smallholder potato farmers predominantly rely on traditional irrigation methods such as furrow irrigation, coupled with excessive irrigation and fertilization to pursue high yields. These practices result in inefficient water and nutrient utilization, resource wastage, and soil structure degradation (Wang et al., 2018; Yang et al., 2017). Therefore, improving crop water productivity and fertilizer use efficiency is critical for ensuring sustainable agriculture in water-scarce regions like northwestern China (Zhang et al., 2022).

Fertigation (drip irrigation with fertilization) delivers water and nutrients directly to the root zone, improving yield through optimized fertilizer use efficiency and enhanced photosynthetic performance, and has been widely adopted in agricultural practice (Wang et al., 2019; Zhang et al., 2024). Potato exhibits high water demand (Aytekin and Çalışkan, 2024), and its shallow root system—with approximately 80–90% of roots distributed within the top 40 cm of soil—renders it highly sensitive to soil moisture conditions, making it vulnerable to water stress and scarcity (Ahmadi et al., 2011; Djaman et al., 2021; Zhang et al., 2017). Both excessive and insufficient irrigation adversely affect different growth stages of potato, ultimately reducing yield (Wang et al., 2020). Studies indicate that water deficit during tuber initiation and bulking stages negatively impacts tuber size, quantity, and quality (Obidiegwu et al., 2015; Tourneux et al., 2003). Drip irrigation (DI), a globally recognized water-saving technique, is extensively utilized in arid and semi-arid regions. It not only enhances crop yield and quality but also significantly improves water and fertilizer use efficiency, while minimizing surface runoff, evaporative losses, and environmental pollution (Kharche et al., 2010; Tian et al., 2017; Wang et al., 2022a). Additionally, deficit irrigation is a potential water-saving strategy that reconciles limited water supply with crop demand during specific growth stages or entire growing seasons, maintaining yield and quality while increasing water use efficiency (Chen et al., 2025; Parkash et al., 2021).

Fertilization represents another critical approach for stabilizing and enhancing crop yields. Rational fertilization promotes potato growth, thereby improving both yield and quality (Wang et al., 2021). As a nutrient-demanding crop, potato exhibits particularly high requirements for nitrogen (N), phosphorus (P), and potassium (K) (Wilmer et al., 2022). Appropriate supply of these essential nutrients is crucial for quality improvement and yield enhancement, as deficiency in any element leads to reduced productivity. In recent years, while chemical fertilizer application in China has increased annually, the growth rate of grain yield per unit area has progressively declined. Although soil testing and formula fertilization methods have been widely promoted, fertilizer use efficiency remains below 40%. Excessive application of traditional chemical fertilizers not only causes significant economic losses but also triggers various environmental issues, including soil acidification, water eutrophication, and water resource pollution (Wang et al., 2022b). Consequently, accelerating the development and application of novel fertilizers has become an inevitable trend in global fertilizer industry development. Fulvic acid (FA), a low-molecular-weight bioactive organic substance, is readily absorbed by plant roots and leaves (Jiao et al., 2024). As a natural biostimulant, it not only optimizes plant nutritional status and leaf pigments but also activates microbial activity (Korsakov et al., 2023), thereby enhancing crop productivity and iron uptake (Turan et al., 2022). Research demonstrates that FA improves yield, quality, and stress resistance in crops. It optimizes root growth, regulates physiological and biochemical responses, improves soil environment and nutrient availability/absorption, enhances stress resistance, and increases soil water retention capacity (Abdelrasheed et al., 2021; El-Mekser et al., 2015; Mauromicale et al., 2011; Mosaad et al., 2024; Roudgarnejad et al., 2022). Furthermore, FA has been shown to significantly promote growth and development in other crops such as onion (Mahmoud et al., 2019), safflower (Moradi et al., 2017), pepper (Aminifard et al., 2012) and lemon (He et al., 2022). Therefore, developing novel FA-containing specialized formulated fertilizers for potato holds significant value for agricultural and ecological systems, representing an important technical approach for improving potato quality and yield while enhancing soil environment.

The combined application of water and fertilizer has been demonstrated to enhance both water-fertilizer use efficiency and potato yield. Numerous studies have confirmed that deficit irrigation (10-30% reduction) coupled with reduced fertilizer application can simultaneously increase yield and improve resource use efficiency (Shrestha et al., 2023). Wang et al. (2019) systematically investigated the effects of irrigation frequency (IF), irrigation level (IL), and fertilization level (FL) on potato growth, yield, quality, and water-fertilizer productivity. Their results revealed that irrigation water use efficiency (IWUE) decreases with increasing water supply, highlighting the importance of optimal irrigation management. Zhang et al. (2022) investigated the interactive effects of irrigation volume and potassium application rate on potato yield, quality, and water productivity. Their study revealed significant coupling effects between irrigation and potassium fertilization on multiple physiological and yield parameters, including Chlorophyll content, dry matter accumulation, tuber yield, reducing sugar content, potassium use efficiency.

Extensive research has been conducted to address water scarcity and low fertilizer use efficiency in arid Northwest China, yet most studies have focused solely on optimizing either irrigation or fertilization strategies, primarily examining the effects of single-factor water-fertilizer coupling on potato growth, yield, economic benefits, and soil nutrients. However, information remains lacking regarding how novel specialized water-soluble fertilizers affect potato growth, photosynthetic performance, yield, and water-fertilizer use efficiency under varying drip irrigation regimes. To address this gap, a novel fulvic acid-incorporated fertilizer was developed by determining component ratios based on target yield nutrient uptake, critical growth stage fertilization requirements, economic feasibility, and fertilizer reduction efficiency. This study aims to (1) Investigate the effects of the new fertilizer on potato growth and tuber yield under different irrigation levels, (2) Analyze water-fertilizer use efficiency across various water-fertilizer management patterns, and (3) Identify optimal management practices through principal component analysis of quantitative data. The research ultimately seeks to establish the best irrigation-fertilization combination for simultaneously enhancing yield, quality, and resource use efficiency in drip-irrigated potato systems, thereby providing scientifically validated management strategies for arid regions like Northwest China.

## 2 Materials and methods

### 2.1 Fertilizer research and development

A novel specialized water-soluble fertilizer containing fulvic acid (F1, F2, F3) was developed specifically for potato cultivation in the arid saline-alkaline regions of Xinjiang. The core raw material, mineral-derived fulvic acid (FA), is rich in active functional groups such as carboxyl and phenolic hydroxyl groups, which contribute to its excellent chelating and buffering capacities—key mechanisms for enhancing nutrient use efficiency. During production, monoammonium phosphate, potassium hydroxide, and liquid nitrogen fertilizer are first added to water in specific proportions to react and form inorganic nutrients. After cooling, liquid nitrogen fertilizer, concentrated fulvic acid solution, concentrated amino acid solution, and boric acid are sequentially added. Subsequently, pre-dissolved potassium formate and urea phosphate are mixed uniformly. Finally, functional additives are incorporated, and the mixture is stirred thoroughly to obtain the final novel fertilizer product. The key to this process lies in controlling the reaction temperature and the sequence of addition, which maximizes the retention of biological activity of each component. The NPK ratio is designed to meet the nutritional requirements of potato growth, resulting in a comprehensive organic–inorganic liquid compound fertilizer suitable for potato cultivation.

### 2.2 Study area description

The field experiment was conducted at the National Soil Quality Akesu Observation and Experiment Station in Baicheng County, Aksu Prefecture, Xinjiang (81°55’E, 41°48’N) starting on May 6, 2023. The experimental site, characterized by a temperate continental arid climate with cold winters and cool summers, is suitable for potato cultivation. It had an average growing season temperature of 22 °C, a total precipitation of 43.2 mm, and a mean monthly humidity of 53.06%, with daily climate data obtained from the Baicheng Meteorological Station. Before planting, soil samples (0–20 cm depth) were collected and analyzed by the Institute of Agricultural Resources and Environment, Xinjiang Academy of Agricultural Sciences, revealing loam-textured soil with moderate fertility: water-soluble salt content 3.4 g·kg^-1^, available nitrogen 109.9 mg·kg^-1^, organic matter 22 g·kg^-1^, available phosphorus 49.9 mg·kg^-1^, total nitrogen 1.25 g·kg^-1^, total phosphorus 1.53 g·kg^-1^, total potassium 11.1 g·kg^-1^, and pH 8.21. Soil field capacity (20.82%) and bulk density (1.31 g·cm^-3^) were determined using the core method before planting.

### 2.3 Experimental design

The potato crop was planted on May 6, 2023, and harvested on August 30 of the same year. The experiment utilized the potato cultivar ‘Xisen 6’, which was co-developed by the National Potato Engineering Technology Research Center and relevant enterprises in Laoling. This medium-to-late maturing cultivar is suitable for both fresh consumption and processing. It exhibits broad adaptability, high yield, superior quality, strong disease resistance, and notable tolerance to saline-alkaline conditions (Xu et al., 2025). The experimental design incorporates two controlled factors: irrigation amount (W) and fertilization rate (F), with drip irrigation arranged in a split-plot design and fertilization treatments implemented using a randomized complete block design. Three irrigation levels were implemented: conventional local practice (W1: 4582.5 m^3^·ha^-1^), 15% deficit (W2: 3865.5 m^3^·ha^-1^), and 30% deficit (W3: 3165 m^3^·ha^-1^). Five fertilization treatments were applied: no fertilizer (CK), conventional fertilization (CF), and three specialized formula fertilizers - F1 (fulvic acid: 120 g·L^-1^; N-P-K=110-100-120), F2 (fulvic acid: 60 g·L^-1^; N-P-K=130-120-150), and F3 (fulvic acid: 30 g·L^-1^; N-P-K=170-150-130). The experiment comprised 15 treatments with three replications each (total 45 plots), with individual plot dimensions of 38.5 m^2^ (11×3.5 m). Potatoes were planted in single-row ridges (40 cm height, 80 cm width) with 110 cm row spacing and 15 cm plant spacing, achieving a planting density of 69,000 plants·ha^-1^. One-meter buffer zones separated irrigation treatments to prevent cross-contamination. Precision irrigation was controlled by flow meters and individual valves per plot, with fertilizer solutions administered through venturi injectors into the drip irrigation system (one fertilizer tank per plot). Drip tapes were laid along ridges, delivering dissolved fertilizers during irrigation events, while standard agronomic practices were maintained throughout the growing season.

### 2.4 Irrigation and fertilization management

A uniform basal fertilizer application was implemented before planting. Fertilizer topdressing was applied through fertigation during four critical growth stages of potato development: seedling stage, bud formation stage, tuber bulking stage, and starch accumulation stage. The CF treatment received a total seasonal fertilizer application of 240 kg·ha^-1^ urea (46.4% N), 240 kg·ha^-1^ diammonium phosphate (18% N, 46% P), 375 kg·ha^-1^ potassium sulfate (50% K), and 225 kg·ha^-1^ compound fertilizer (17%N, 17%P, 17%K), with stage-specific application ratios of 5:2:1:0 (urea), 1:1:1:1 (diammonium phosphate), 7:6:6:6 (potassium sulfate), and 0:1:1:1 (compound fertilizer) across the four growth stages (seedling, bud formation, tuber bulking, and starch accumulation). The F1, F2, and F3 treatments each received seasonal fertilizer applications consisting of 75 kg·ha^-1^ urea (46.4% N), 15 kg·ha^-1^ diammonium phosphate (18% N, 46% P), 75 kg·ha^-1^ potassium sulfate (50% K), and 750 kg·ha^-1^ of their respective fulvic acid-enriched water-soluble fertilizers (F1,F2,F3). The application ratios were 5:0:0:0 for urea, 1:0:0:0 for diammonium phosphate, 5:0:0:0 for potassium sulfate, and 2:3:3:2 for the novel fertilizers across the four growth stages. The complete nutrient compositions (N, P, K) for each fertilizer treatment are presented in **Table 1**.

**Table 1:** Total nutrient content of nitrogen, phosphorus, and potassium across fertilization treatments.

Treatment	Fulvic acid (g·L^-1^)	N (kg·ha^-1^)	P (kg·ha^-1^)	K (kg·ha^-1^)
CK	0	0	0	0
CF	0	258.5	245.3	338.3
F1	100	173.6	161.1	212.2
F2	60	185.1	172.7	236.6
F3	30	208.2	190.1	225.0

During the potato growing season, irrigation was applied five times corresponding to key growth stages: seedling stage, bud formation stage, tuber bulking stage, starch accumulation stage, and maturation. The W1 treatment received 1365m^3^·ha^-1^, 877.5 m^3^·ha^-1^, 1170 m^3^·ha^-1^, 877.5 m^3^·ha^-1^ and 292.5 m^3^·ha^-1^at each respective growth stage, Irrigation amounts for the W2 treatment were 1150.5 m^3^·ha^-1^, 742.5 m^3^·ha^-1^, 975 m^3^·ha^-1^, 742.5 m^3^·ha^-1^ and 255 m^3^·ha^-1^, while the W3 treatment received 960 m^3^·ha^-1^, 600 m^3^·ha^-1^, 810 m^3^·ha^-1^, 600 m^3^·ha^-1^, and 195 m^3^·ha^-1^ at the corresponding developmental phases.

### 2.5 Parameter measurements

(1) Growth parameter measurements: For each treatment, three plants were randomly sampled from each of the three replicate plots (total nine plants per treatment). Plant height, stem diameter, and aboveground dry matter accumulation were determined during the tuber bulking stage. Plant height was measured using a tape measure to the nearest 1 cm. Stem diameter was measured at the soil-stem interface with a vernier caliper (precision: 0.1 mm). The sampled plants were carefully cleaned of surface contaminants, washed, and then surface-dried with absorbent paper. The aboveground biomass was oven-dried at 105 °C for 30 minutes (enzyme deactivation), followed by drying at 75 °C to constant weight. Dry weight measurements were obtained using an analytical balance (precision: 0.1 g) and averaged across replicates.

(2) Photosynthetic characterization: During the tuber bulking stage (July 20, 9:00-10:00 AM), three plants were randomly sampled from each of the three replicate plots (total nine plants per treatment). For each plant, three healthy leaves at identical nodal positions were chosen for photosynthetic measurements using a TP-3051D portable photosynthesis system (Zhejiang Top Cloud-Agri Technology Co., Ltd., Hangzhou, China). The following parameters were recorded: net photosynthetic rate (Pn), transpiration rate (Tr), stomatal conductance (Gs), and intercellular CO_2_ concentration (Ci), with triplicate measurements per leaf. The largest leaf from each plant was selected for chlorophyll assessment, where SPAD values (relative chlorophyll content) were determined at three distinct points using a chlorophyll meter. Mean values of all measurements were calculated for statistical analysis.

(3) The yield and yield components of potatoes were determined as follows: At maturity, all plants within each plot were uniformly harvested for total yield measurement, with the final yield subsequently calculated. Additionally, three randomly selected plants per experimental plot were sampled to evaluate yield parameters. The weight of tubers per plant and the number of tubers per plant were recorded. Marketable tuber yield (defined as tubers weighing >75 g per individual) and the number of marketable tubers per plant were also determined, followed by the calculation of the marketable rate.

(4) Soil moisture content measurement: At the tuber bulking stage, soil moisture content in the 0–60 cm soil layer was measured using a soil auger. The collected soil samples were placed in aluminum boxes, weighed, and then dried in an oven at 105 °C until they reached a constant weight. After drying, the samples were reweighed to calculate the soil moisture content.


(1) $$W=\;\frac{M2-M}{M1-M2}\;\times \;100\%$$

In the above equation,W represents soil moisture content (%), M1 is the total mass of fresh soil and the aluminum box (g), M2 is the total mass of dry soil and the aluminum box (g), and M is the mass of the aluminum box (g) (Chadwick et al., 2018).

(5) The crop water consumption (ETc) is determined using the water balance equation (Ierna and Mauromicale, 2018):


(2) ETc=P+I+Cr−Dp−Rf±ΔS

where P is precipitation; I is the depth of irrigation water applied; Cr is capillary rise; Dp is deep percolation, Rf is the run off and ΔS is the change in soil moisture content, with all terms expressed in mm. The amount of water obtained from capillary rise was negligible. Due to the considerable depth of groundwater (2–3 meters), the influence of Dp is considered negligible. Since soil water storage changes minimally below 90 cm, variations beyond this depth are also deemed insignificant. Additionally, no surface runoff occurs in the experimental fields as field ridges border all plots. As Cr, Dp and R were negligible, ETc was calculated as:


(3) ETc = P + I ±Δ S

(6) Calculation of water use efficiency (WUE, kg·m^-3^) and irrational water use efficiency (IWUE, kg·m^-3^):

The formula for WUE (kg·m^-3^) is as follows:


(4) $$WUE=\frac{Y}{ETc}\;$$

The formula for IWUE (kg·m^-3^) is as follows:


(5) $$IWUE=\frac{Y}{I}$$

where Y=crop yield (kg·ha^-1^), ETc=total water consumption (m^3^·ha^-1^), I=total irrigation amount (m^3^·ha^-1^).

(7) Calculation of Fertilizer Partial Factor Productivity (PFP, kg·kg^-1^).

The formula for PFP is as follows:


(6) $$PFP=\frac{Y}{\text{F}}$$

where Y=crop yield (kg·ha^-1^), F=total amount of fertilizer applied during the entire growing season (kg·ha^-1^).

### 2.6 Statistical analyses

The experimental data are organized using Microsoft Excel 2010. A two-way ANOVA, Pearson correlation analysis, and principal component analysis are performed using SPSS Statistics 27.0. Multiple comparisons of significant effects are conducted using Tukey’s HSD post hoc test, with statistical significance defined at the p < 0.05 level. Figures are generated using Origin 2021 and the ChiPlot platform (https://www.chiplot.online/).

## 3 Results

### 3.1 Effects of different water-fertilizer regimes on potato growth

**Figure 1:**
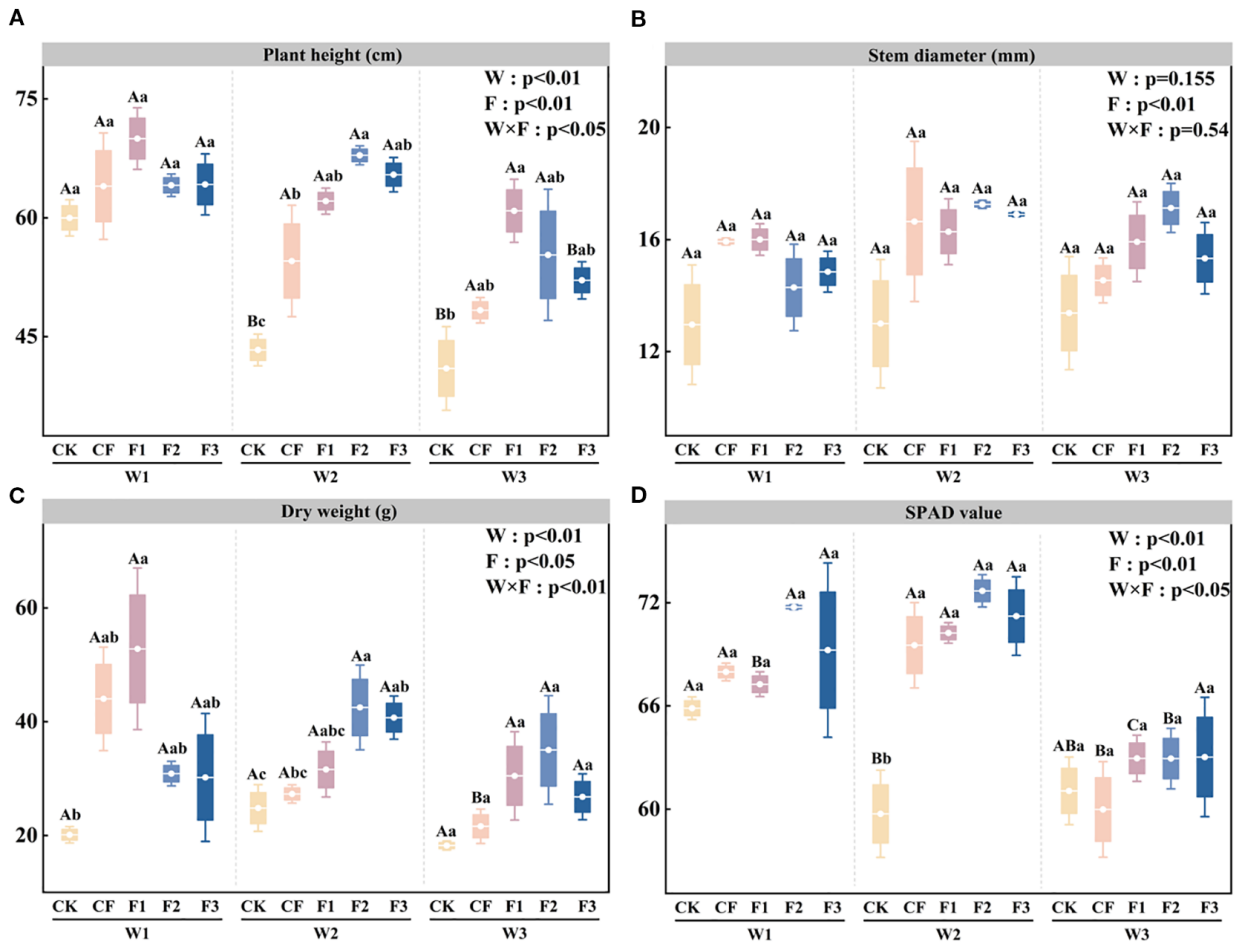
Plant height (PH; **A**), stem diameter (SD; **B**), dry weight (DW; **C**), and SPAD value (SPAD; **D**) under different irrigation levels and fertilizer treatments. Bars represent means ± standard error. Different lowercase letters above the bars indicate significant differences (p < 0.05) among fertilizer treatments under the same irrigation level, while different uppercase letters denote significant differences (p < 0.05) among irrigation levels under the same fertilizer treatment, as determined by Tukey’s HSD test. Irrigation regimes: W1 (conventional practice, 4582.5 m^3^·ha^−1^), W2 (15% deficit, 3865.5 m^3^·ha^−1^), W3 (30% deficit, 3165 m^3^·ha^−1^). Fertilizer treatments: CK (no fertilizer), CF (conventional fertilizer), F1 (fulvic acid: 120 g·L^−1^; N–P–K = 110–100–120), F2 (fulvic acid: 60 g·L^−1^; N–P–K = 130–120–150), F3 (fulvic acid: 30 g·L^−1^; N–P–K = 170–150–130).

The application of a novel, specialized fertilizer under different drip irrigation systems is demonstrated to enhance aboveground potato growth during the tuber bulking stage significantly. ANOVA results (**Figure 1A**) reveal that irrigation level (W), fertilization treatment (F), and their interaction (W×F) exert significant effects on plant height. Within identical fertilization treatments, plant height increases progressively with irrigation volume, peaking under the W1 treatment. Specifically, W3F3 shows reductions of 18.86% and 20.37% compared to W1F3 and W2F3, respectively. Under the W2 irrigation regime, the W2F2 treatment shows superior performance, exhibiting enhancements of 56.67% and 24.44% relative to W2CK and W2CF, respectively. Analysis of **Figure 1B** reveals that stem diameter is significantly influenced by fertilizer treatment (F), while neither irrigation level (W) nor the interaction between W and F has a significant effect. The maximum stem diameter values, ranging from 13.00 to 17.26 mm, are consistently observed under the W2 irrigation regime. Analysis of **Figure 1C** shows that aboveground dry weight is significantly influenced by irrigation level (W), fertilization treatment (F), and the interaction between W and F. Compared to the CK, the formulated fertilizer treatments (F1-F3) increase dry matter accumulation by 50.03-162.4%, 27.33-71.24%, and 46.97-92.18% under W1, W2, and W3 irrigation regimes, respectively. Notably, the F2 treatment under W2 irrigation demonstrates significant improvements of 71.24% and 55.72% compared to CK and CF, respectively. Analysis of **Figure 1D** reveals that the relative chlorophyll content (SPAD values) is significantly affected by irrigation level (W), fertilization treatment (F), and the interaction between W and F. Under the W2 irrigation regime, SPAD values in the novel fertilizer treatments are significantly higher than those in W2CK, showing increases of 17.6-21.69% compared to the control. When comparing treatments with identical fertilization levels, W1F2 and W2F2 demonstrate significantly greater SPAD values than W3F2, while W3F1 exhibits reductions of 6.4% and 10.38% relative to W1F1 and W2F1, respectively. These results clearly indicate that water deficit significantly impairs chlorophyll accumulation in potato leaves under equivalent fertilization conditions. The above results suggest that an appropriate combination of water and fertilizer can significantly promote potato shoot growth and chlorophyll accumulation, thereby establishing a strong physiological foundation for subsequent photosynthesis and yield formation.

### 3.2 Effects of different water-fertilizer regimes on leaf photosynthetic rate in potato

**Figure 2:**
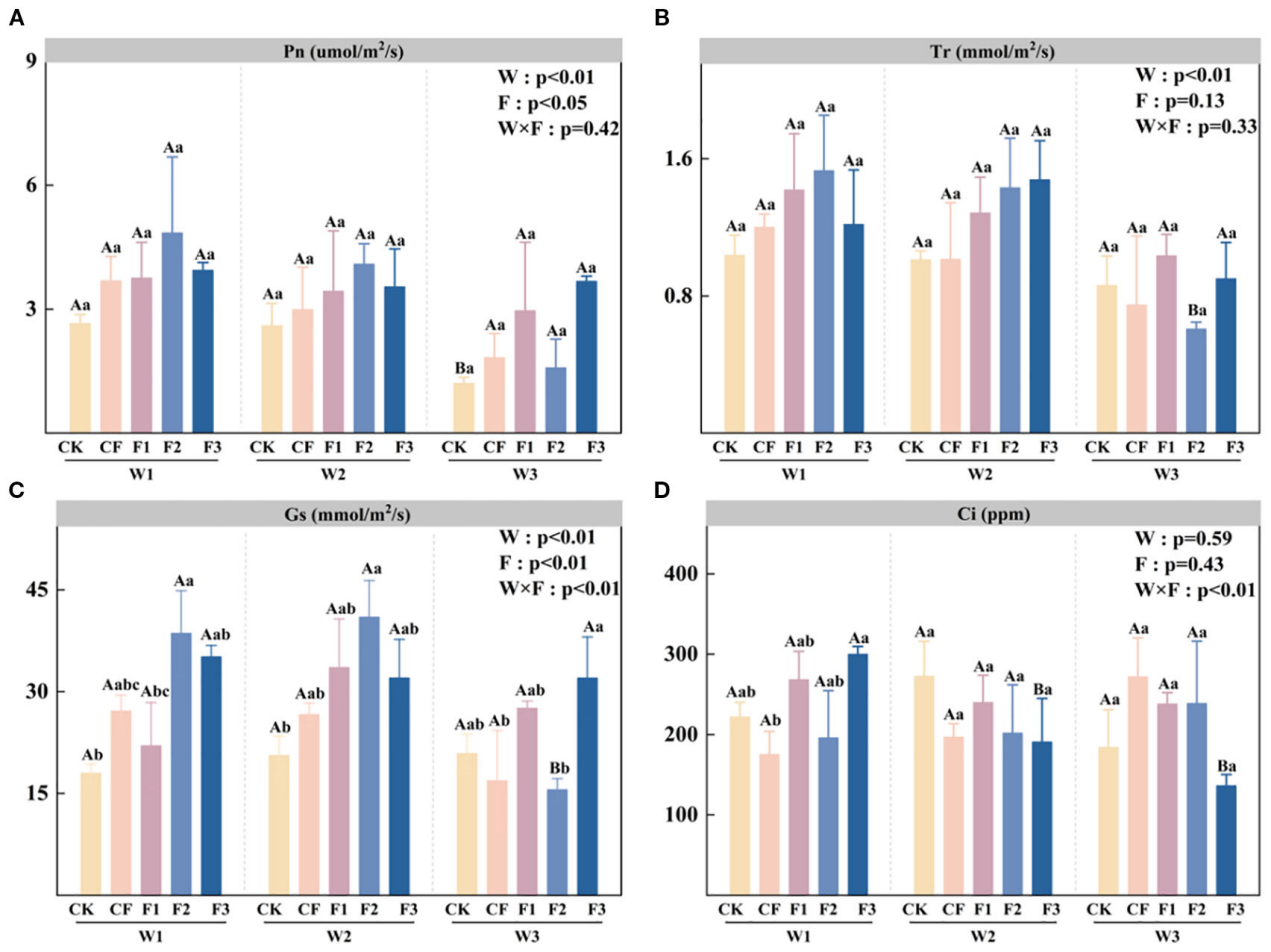
Net photosynthetic rate (Pn; **A**), transpiration rate (Tr; **B**), stomatal conductance (Gs; **C**), and intercellular CO_2_ concentration (Ci; **D**) under different irrigation levels and fertilizer treatments. Bars represent means ± standard error. Different lowercase letters above the bars indicate significant differences (p < 0.05) among fertilizer treatments under the same irrigation level, while different uppercase letters denote significant differences (p < 0.05) among irrigation levels under the same fertilizer treatment, as determined by Tukey’s HSD test. Irrigation regimes: W1 (conventional practice, 4582.5 m^3^·ha^−1^), W2 (15% deficit, 3865.5 m^3^·ha^−1^), W3 (30% deficit, 3165 m^3^·ha^−1^).Fertilizer treatments: CK (no fertilizer), CF (conventional fertilizer), F1 (fulvic acid: 120 g·L^−1^; N–P–K = 110–100–120), F2 (fulvic acid: 60 g·L^−1^; N–P–K = 130–120–150), F3 (fulvic acid: 30 g·L^−1^; N–P–K = 170–150–130).

Analysis of **Figure 2A** demonstrates that net photosynthetic rate (Pn) is significantly influenced by both irrigation level (W) and fertilization treatment (F). Similarly, **Figure 2B** shows that the transpiration rate (Tr) is significantly affected by the irrigation level (W). Under the F2 fertilization treatment, W3 irrigation results in significantly lower Tr values compared to W1 and W2, with reductions of 60.35% and 57.58% observed in W3F2 relative to W1F2 and W2F2, respectively. ANOVA results in **Figure 2C** indicate that stomatal conductance (Gs) is significantly affected by irrigation level (W), fertilization treatment (F), and the interaction between W and F. Under both W1 and W2 irrigation regimes, the F2 treatment demonstrates significantly higher Gs values than CK, with increases of 114.74% and 99.14%, respectively. However, under water-deficit irrigation (W3), the F2 treatment exhibits the lowest stomatal conductance (Gs), showing a 25.52% reduction compared to CK. In contrast, the F3 treatment demonstrates optimal performance under these conditions, with Gs values 53.45% and 89.98% higher than CK and CF, respectively. When examining the F2 fertilization treatment specifically, water deficit (W3) causes significant reductions in stomatal conductance, decreasing by 59.74% and 62.12% relative to W1F2 and W2F2 treatments. The interaction between irrigation and fertilization (W×F) has a significant effect on intercellular CO_2_ concentration (Ci). Under equivalent irrigation levels, most novel fertilizer treatments show lower Ci values compared to CK. However, these differences are not statistically significant, except for W1F3, which exhibits a significantly higher Ci than CF. Within the F3 fertilization treatment, full irrigation (W1) results in significantly greater Ci values compared to both W2F3 and W3F3, with increases of 9.74% and 9.76%, respectively. The improvement in photosynthetic performance further explains the promotive effect of the novel fertilizer on potato growth under moderate deficit irrigation, ensuring an energy supply for subsequent tuber yield and biomass accumulation.

### 3.3 Effects of different water-fertilizer regimes on potato yield and its components

**Figure 3:**
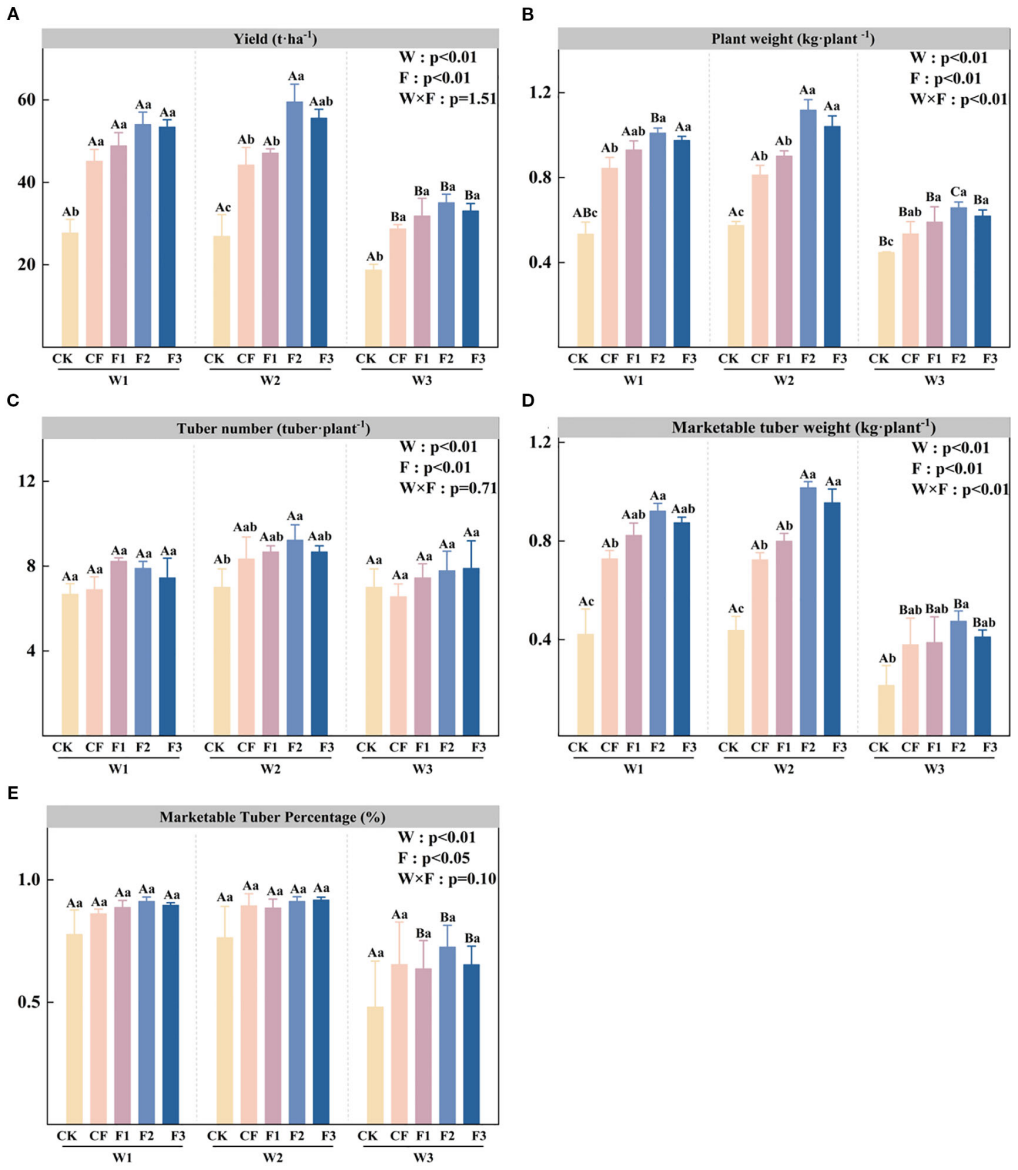
Yield **(A)**, plant weight **(B)**, tuber number **(C)**, marketable tuber weight **(D)**, and marketable tuber percentage **(E)** under different irrigation levels and fertilizer treatments. Bars represent means ± standard error. Different lowercase letters above the bars indicate significant differences (p < 0.05) among fertilizer treatments under the same irrigation level, while different uppercase letters denote significant differences (p < 0.05) among irrigation levels under the same fertilizer treatment, as determined by Tukey’s HSD test. Irrigation regimes: W1 (conventional practice, 4582.5 m^3^·ha^−1^), W2 (15% deficit, 3865.5 m^3^·ha^−1^), W3 (30% deficit, 3165 m^3^·ha^−1^).Fertilizer treatments: CK (no fertilizer), CF (conventional fertilizer), F1 (fulvic acid: 120 g·L^−1^; N–P–K = 110–100–120), F2 (fulvic acid: 60 g·L^−1^; N–P–K = 130–120–150), F3 (fulvic acid: 30 g·L^−1^; N–P–K = 170–150–130).

The enhancement of growth indicators, coupled with improved photosynthetic performance, collectively contributes to a significant increase in yield. Analysis of **Figure 3A** demonstrates that potato yield is significantly affected by both irrigation level (W) and fertilization treatment (F), with the three novel formula fertilizers consistently showing significant yield increases across all irrigation regimes - achieving 76.54-95.25% (W1), 75.13-121.29% (W2), and 70.32-87.56% (W3) improvements compared to control. The W2F2 treatment achieves optimal yield performance (59.49 t·ha^-1^), demonstrating a 121.29% increase over W2CK and a 26.36% yield advantage compared to W2F1. Under equivalent fertilization conditions, the water-deficit irrigation treatment (W3) yields significantly lower than those of both W1 and W2 treatments. **Figure 3B** shows that tuber yield per plant is significantly influenced by irrigation level (W), fertilization treatment (F), and the interaction between W and F. Under identical irrigation regimes, all three novel fertilizer formulations significantly enhance single plant weight, with F2 consistently outperforming other treatments, followed by F3. When examining equivalent fertilization levels, water-deficit irrigation (W3) yields are significantly reduced compared to both W1 and W2. The W2 irrigation regime produces the highest tuber yield per plant among all treatments, with the W2F2 combination demonstrating a 69.77% significant increase compared to W3F2. Analysis of **Figure 3D** reveals that the marketable tuber yield per plant is significantly affected by irrigation level (W), fertilization treatment (F), and their interaction (W×F). Under both W1 and W2 irrigation regimes, application of the three novel fertilizer formulations significantly enhances marketable yield, with F2 treatment demonstrating superior performance and showing statistically significant increases over CK. Furthermore, under identical fertilization treatments, marketable yields under W1 and W2 irrigation are significantly higher than those under W3, clearly indicating that water deficit substantially reduces marketable tuber production. Analysis of **Figure 3E** indicates that the marketable tuber ratio is significantly influenced by irrigation level (W), fertilization treatment (F), and their interaction (W × F). For all three formula fertilizer treatments (F1, F2, and F3), water-deficit irrigation (W3) results in significantly lower marketable ratios, compared to reductions of 20.46-28.24% relative to W1 and 20.47-28.78% relative to W2, demonstrating that water stress significantly impairs potato marketability. Analysis of yield and its components indicates that the synergistic regulation of water and fertilizer not only directly affects tuber formation but also influences yield per plant and the marketable tuber rate, highlighting the critical role of water and fertilizer management in potato production.

### 3.4 Water and fertilizer use efficiency under different water-fertilizer regimes

**Figure 4:**
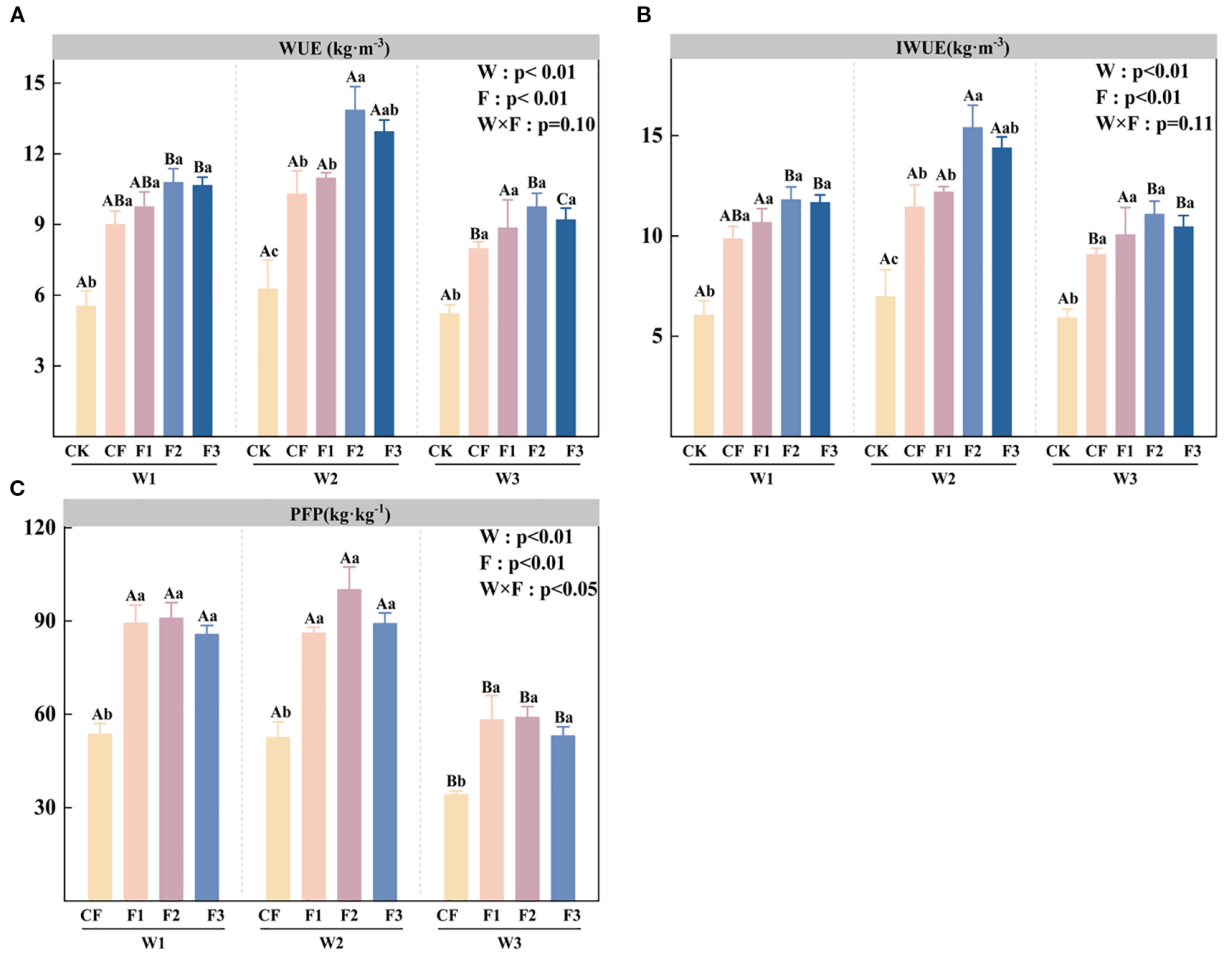
Water use efficiency (WUE; **A**), irrigation water use efficiency (IWUE; **B**), and partial factor productivity (PFP; **C**) under different irrigation levels and fertilizer treatments. Bars represent means ± standard error. Different lowercase letters above the bars indicate significant differences (p < 0.05) among fertilizer treatments under the same irrigation level, while different uppercase letters denote significant differences (p < 0.05) among irrigation levels under the same fertilizer treatment, as determined by Tukey’s HSD test. Irrigation regimes: W1 (conventional practice, 4582.5 m^3^·ha^−1^), W2 (15% deficit, 3865.5 m^3^·ha^−1^), W3 (30% deficit, 3165 m^3^·ha^−1^).Fertilizer treatments: CK (no fertilizer), CF (conventional fertilizer), F1 (fulvic acid: 120 g·L^−1^; N–P–K = 110–100–120), F2 (fulvic acid: 60 g·L^−1^; N–P–K = 130–120–150), F3 (fulvic acid: 30 g·L^−1^; N–P–K = 170–150–130).

Water use efficiency under different water and fertilizer treatments was calculated using Equations 1–4. **Figure 4A** demonstrates that water use efficiency (WUE) is significantly influenced by irrigation level (W) and fertilization treatment (F) (p < 0.05). Under the W1, W2, and W3 irrigation treatments, the application of the new fertilizer significantly increased WUE by 76.54%–95.25%, 75.13%–121.29%, and 70.32%–87.56%, respectively, compared to the CK. Furthermore, under equivalent fertilization conditions, W2 irrigation consistently produced higher WUE than W3 irrigation, as demonstrated by W2F2’s 28.46% and 42.06% advantages over W1F2 and W3F2, respectively, and W2F3’s 21.38% and 40.68% superior performance compared to W1F3 and W3F3. Irrigation water use efficiency under different water and fertilizer treatments was calculated using Equation 5. Analysis of **Figure 4B** reveals that irrigation water use efficiency (IWUE) is significantly affected by both irrigation level (W) and fertilization treatment (F). Under identical irrigation conditions, the three novel formula fertilizers (F1-F3) significantly enhance IWUE compared to the CK. The W2F2 treatment demonstrates optimal IWUE performance, showing a 121.3% increase over W2CK, followed by W2F3 with a 106.67% improvement relative to CK. When examining equivalent fertilization levels, the W2 irrigation regime consistently yields a higher IWUE than the W3 regime. Partial factor productivity under different water and fertilizer treatments was calculated using Equation 6. Analysis of **Figure 4C** indicates that the partial factor productivity (PFP) is significantly influence by irrigation level (W), fertilization treatment (F), and their interaction (W×F). Under identical irrigation conditions, the novel fertilizer formulations (F1-F3) demonstrate significantly higher PFP compared to conventional fertilization (CF), with increases ranging from 59.99-69.74% (W1), 64-90.22% (W2), and 55.65-73.06% (W3). The W2F2 treatment achieves maximum PFP, showing a 90.66% improvement over W2CF. When comparing irrigation levels within the same fertilization treatment, the W2 regime consistently produces the highest PFP values, while water deficit (W3) results in significant reductions of 34.86-38.11% and 32.39-41.08% compared to W1 and W2, respectively. The enhancement of water and fertilizer use efficiency demonstrates that the application of the novel fulvic acid-based fertilizer under moderate water-saving conditions facilitates efficient resource utilization, thereby providing a technical strategy for sustainable potato production in arid regions.

### 3.5 Correlation and principal component analysis of key growth stage indicators in potato

**Figure 5:**
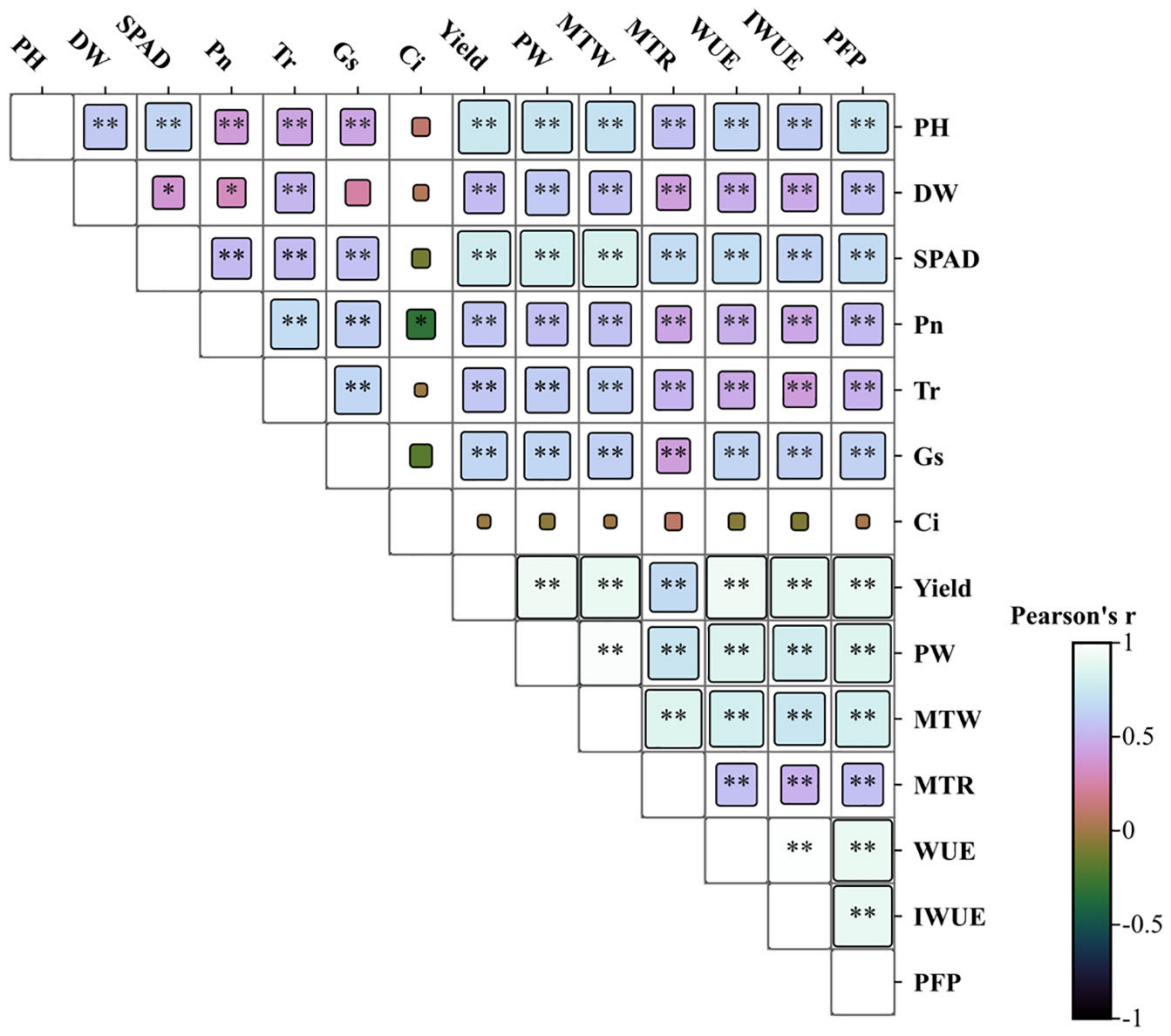
Correlation analysis of key potato parameters under different water and fertilizer treatments. * and ** indicate significant differences at p < 0.05, and p < 0.01. PH, plant height; SD, stem diameter; DW, aboveground dry weight; SPAD, relative chlorophyll content; Pn, net photosynthetic rate; Tr, transpiration rate; Gs, stomatal conductance; Ci, intercellular carbon dioxide concentration; Yield, Total yield per acre; PW, plant weight; MTW, marketable tuber weight per plant and; MTR, marketable tuber percentage; WUE, water use efficiency; IWUE, irrigation water use efficiency; PFP, partial factor productivity.

**Figure 6:**
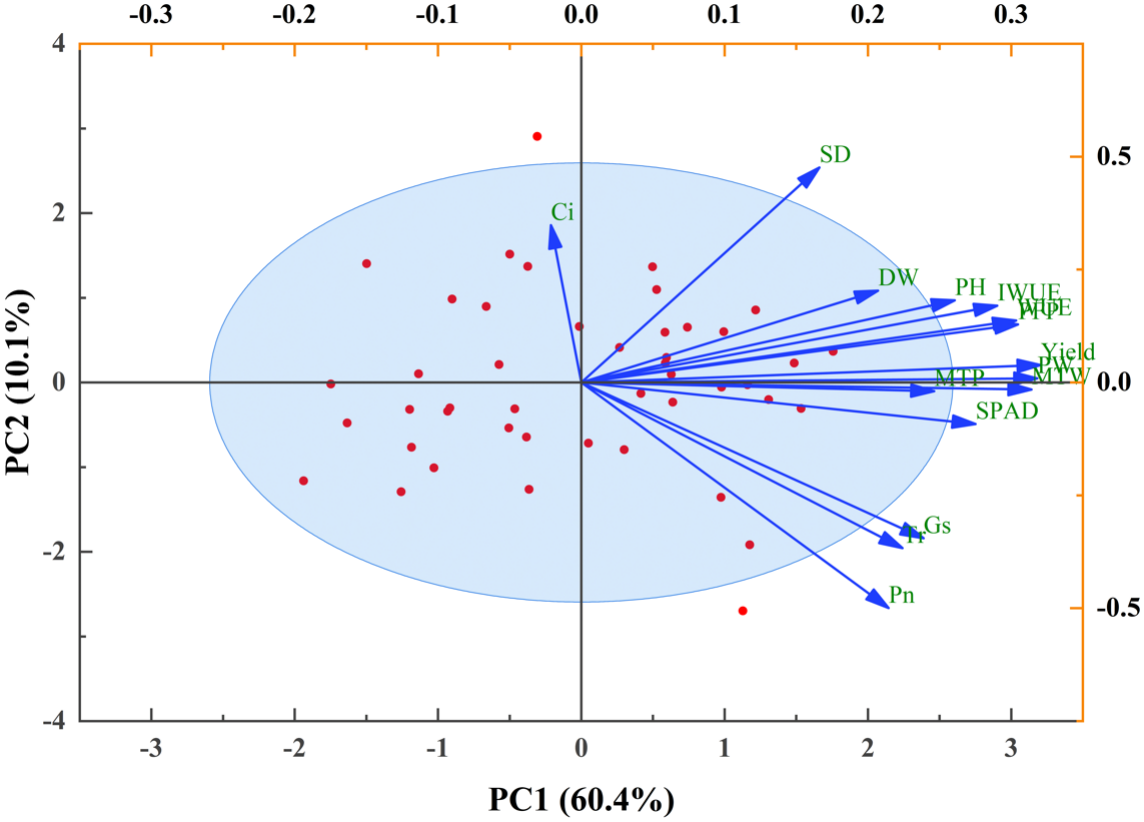
Principal component analysis of potato growth, yield components, and water-fertilizer use efficiency under different water-fertilizer regimes.

A correlation analysis was performed to examine the relationships among key traits in potato during the tuber bulking stage. The results unveiled a network of significant correlations linking canopy growth, photosynthetic parameters, yield components, and efficiencies of water and fertilizer utilization (**Figure 5**). Plant height (PH), aboveground dry weight (DW), and SPAD values show highly significant positive correlations with both total yield and yield components (p<0.01). Net photosynthetic rate (Pn) demonstrates strong positive associations with transpiration rate (Tr), stomatal conductance (Gs), and yield parameters (p<0.01), while exhibiting a significant negative correlation with intercellular CO_2_ concentration (Ci) (p<0.05). Conversely, Ci is negatively correlated with growth indicators and yield components (p<0.05). Furthermore, both water use efficiency (WUE) and partial factor productivity of fertilizer (PFP) exhibit highly significant positive correlations with PH, DW, SPAD, Pn, and all yield-related parameters (p < 0.01). Principal component analysis (PCA) was employed to evaluate and optimize water-fertilizer management strategies for potato cultivation in northwest China, focusing on tuber yield, quality, water use efficiency (WUE), and fertilizer productivity (**Figure 6**). The comprehensive evaluation scores, calculated through PCA (**Table 2**), demonstrate that under identical irrigation regimes, novel fertilizer treatments consistently outperform both control (CK) and conventional fertilization (CF). Among these, the F2 formula fertilizer shows superior performance, with the top three ranked treatments being W2F2 > W2F3 > W1F1, while W3CK ranks lowest. These results indicate that under the experimental conditions, the combination of W2 irrigation level with either F2 or F3 fertilizers represents the optimal approach for simultaneously enhancing potato yield and improving water-fertilizer use efficiency.

**Table 2:** Comprehensive evaluation of potato growth, yield components, and water-fertilizer use efficiency under varied water-fertilizer treatments based on principal component analysis.

Irrigation levels	Fertilization levels	Comprehensive evaluation	Ranking
W1	CK	-1.14	14
	CF	0.01	9
	F1	0.54	3
	F2	0.16	8
	F3	0.17	7
W2	CK	-1.04	13
	CF	0.25	6
	F1	0.52	4
	F2	1.23	1
	F3	0.95	2
W3	CK	-1.11	15
	CF	-0.61	12
	F1	-0.07	10
	F2	0.29	5
	F3	-0.16	11

## 4 Discussion

### 4.1 Effects of water-fertilizer regimes on potato growth

Optimal water-fertilizer management is critical for potato growth and development. As irrigation levels decrease, plant height, aboveground dry matter accumulation, and relative chlorophyll content (SPAD) are consistently suppressed, likely due to water stress impairing root functionality and consequently reducing nutrient and water uptake capacity (Feng et al., 2022). In this study, under full irrigation (W1), novel fertilizer treatments achieve maximum plant height, showing no significant difference from W2 treatments. This finding is consistent with the results reported by Wang et al. (2019) indicating that the F1 and F2 fertilization treatments can mitigate the impact of water stress on potato growth. It is hypothesized that fulvic acid (FA) may function either as an antioxidant scavenging excess reactive oxygen species (ROS) or as a signaling molecule that induces the production of antioxidants, thereby reducing oxidative damage in plant cells and consequently enhancing drought resistance. Further investigation into related plant physiological indicators is warranted in future studies (Song et al., 2025). In contrast, under the W3 treatment, a significant accumulation of ROS and malondialdehyde (MDA) was observed in plants, leading to pronounced oxidative damage. Furthermore, the lower effective content of FA and potassium in the F3 treatment may have directly limited its efficacy in helping potatoes cope with drought stress (Chen et al., 2022). While novel fertilizer applications consistently promote aboveground dry matter accumulation under both full (W1) and moderate (W2) irrigation regimes, their efficacy is shown to be moisture-dependent, as evidenced by the greater sensitivity of aboveground biomass to irrigation level (p < 0.01) compared to fertilization (p < 0.05), consequently rendering them ineffective in mitigating the negative impacts of severe water deficit (W3) on dry matter production. The results of this study indicate that treatments W1F1 and W2F3 significantly increase aboveground dry matter accumulation in potatoes compared to the CK treatment. Aboveground dry matter serves as a key indicator of photosynthetic product accumulation (Liang et al., 2022), reflecting not only enhanced plant growth but also a greater capacity for translocating assimilates to the tubers, thereby establishing a material basis for tuber yield and quality formation. Notably, dry matter accumulation and distribution directly affect tuber storage performance. Dry matter content is an important quality trait in potatoes (Wold et al., 2021), with higher levels generally associated with increased starch accumulation in tubers (Carpenter et al., 2015; Turesson et al., 2014; Xue et al., 2019). This starch serves as an energy reserve for sprouting and early plant establishment (Seung et al., 2015).During storage, tubers undergo water evaporation and respiratory loss of dry matter (Li et al., 2022; Smith et al., 2020; Therasme et al., 2021). It is therefore suggested that tubers from the W2F2 treatment have the potential to accumulate higher dry matter, which may delay the rate of dry matter loss during storage, ultimately reducing weight loss and improving sprouting suppression capacity, thereby exhibiting better storability. However, this study did not directly measure physiological changes during tuber storage. Future research should incorporate storage trials to further evaluate the effects of different water and fertilizer management strategies on the postharvest quality and storage stability of potatoes. Zin El-Abedin et al. (2019) reported that mild deficit irrigation maintains relatively high leaf chlorophyll content in potatoes, though not significantly different from full irrigation, which aligns with the present findings where W2 irrigation under identical fertilization achieves peak SPAD values without statistical distinction from W1. This chlorophyll preservation is attributed to optimized water management facilitating enhanced uptake of water and essential nutrients (nitrogen, potassium, magnesium, and iron) that support chlorophyll biosynthesis (Amin et al., 2022), and subsequently improve photosynthetic efficiency. This finding is also consistent with the view reported by Mosaad et al. that fulvic acid (FA) application can enhance chlorophyll content (Mosaad et al., 2025). It is speculated that FA may improve nutrient availability, increase cell membrane permeability, and enhance intracellular signal transduction function, thereby promoting root growth. These improvements subsequently lead to increased chlorophyll content and photosynthetic efficiency, ultimately activating carbon and nitrogen metabolism (Qin et al., 2023; Song et al., 2025; Wang et al., 2023). In contrast, drought stress has been shown to impair water uptake capacity, thereby reducing nutrient acquisition and ultimately decreasing chlorophyll content (Ma et al., 2022), which is consistent with the significantly lower SPAD values observed in the W3 treatment compared to W1 and W2 in the present study.

### 4.2 Effects of water-fertilizer treatments on leaf photosynthetic rate in potato

Water serves as a fundamental substrate for plant photosynthesis (Wang et al., 2019), directly regulating leaf gas exchange and physiological metabolism. Existing studies have indicated that foliar application of FA at appropriate concentrations can significantly enhance chlorophyll content, activities of photosynthesis-related enzymes, and upregulate associated gene expression, thereby increasing the net photosynthetic rate (Pn) in crop seedlings under drought stress (Fang et al., 2020; Liu et al., 2022; Zhu et al., 2022). In the present study, however, no significant differences in Pn were observed across different drip irrigation levels under the same fertilization regime. It is hypothesized that the FA present in each fertilizer treatment alleviated drought-induced damage by mitigating the adverse effects on the maximal photochemical efficiency (Fv/Fm), actual photochemical efficiency (ΦPSII), electron transport quantum yield, and PSII activity, while also helping to maintain chloroplast ultrastructure. As a result, the detrimental impact of drought stress on leaf photosynthesis was reduced, leading to comparable Pn values across irrigation treatments (Yu et al., 2023). The study reveals that deficit irrigation significantly reduces net photosynthetic rate (Pn) in potato plants, while full irrigation maintains higher stomatal conductance (Gs) and transpiration rate (Tr) (Zin El-Abedin et al., 2019). This finding is broadly consistent with the experimental results observed in the present study. This physiological response pattern likely stems from drought-induced stomatal limitations, where water stress triggers stomatal closure to reduce water loss, consequently decreasing Gs and restricting CO_2_ substrate availability for carboxylation. Such stomatal constraints ultimately impair carbon assimilation efficiency, leading to reduced photosynthetic activity and compromised nutrient uptake capacity (Amin et al., 2022; Osakabe et al., 2014). Furthermore, the observed increase in intercellular CO_2_ concentration, despite a reduced transpiration rate and stomatal conductance, suggests that metabolic or biochemical limitations within the mesophyll cells contribute significantly to the decline in net photosynthetic rate. Under severe conditions, drought stress can induce photoinhibition and oxidative stress, which may further exacerbate the loss of photosynthetic capacity (Sarker et al., 2019). However, the present study demonstrates that application of novel fertilizers significantly enhances stomatal conductance, thereby mitigating the inhibitory effects of deficit irrigation on photosynthesis. This protective mechanism is evidenced by the non-significant differences in Pn between W3 and the W1 and W2 when novel fertilizers were applied.

### 4.3 Effects of water-fertilizer management on potato yield and its components

Tuber yield, tuber yield per plant, and marketable tuber percentage represent critical productivity indicators in potato cultivation. Yield is significantly influenced by irrigation levels, with the W2 treatment demonstrating optimal performance, while the W3 treatment substantially reduces productivity. These findings align with Eid’s observations that extreme water deficit markedly decreases evapotranspiration, consequently impairing photosynthesis, stomatal conductance, and transpiration, ultimately leading to reduced biomass accumulation (Eid et al., 2020; Martínez‐Goñi et al., 2023). The shallow root architecture characteristic of potato further exacerbates its sensitivity to water stress (Ojala et al., 1990), as drought conditions limit both canopy development and biomass production through multiple physiological constraints. The W2 irrigation regime achieves optimal tuber quality and the highest marketable tuber production, likely due to its ability to enhance biomass accumulation while improving resource use efficiency (Liu et al., 2006; Sepaskhah and Ahmadi, 2010; Yactayo et al., 2013; Yu et al., 2020). Previous studies have reported that nitrogen application exceeding 224 kg·ha^-1^ fails to significantly enhance crop yield (Makani et al., 2020). In the present study, all novel fertilizer treatments were tested under reduced fertilization regimes, with the conventional fertilization (CF) treatment applying nitrogen above this threshold (224 kg·ha^-1^) and subsequently demonstrating significantly lower potato yields compared to the three novel formulations across all irrigation levels - showing reductions of 7.7-16.55% (W1), 6.12-25.7% (W2), and 9.86-18.15% (W3). The fulvic acid-enriched specialized fertilizers are shown to mitigate the detrimental effects of water stress while positively influencing yield through improved plant water relations and enhanced photosynthetic rates in tuber-bearing potato plants, indicating their critical role in overcoming soil moisture limitations. This effect is attributed to the increased nutrient uptake and translocation to tubers facilitated by the bioavailable fulvic acid components in the novel fertilizers (Wang et al., 2020). Humic acid fertilizer application significantly enhances crop growth and yield (Suntari et al., 2015). This improvement is attributed to stimulated root development and increased nutrient uptake capacity (Calvo et al., 2014). Irrigation level significantly influences potato yield components, with tuber quality initially increasing then decreasing as water application rises, peaking under the W2 treatment which demonstrates optimal performance in tuber yield per plant, marketable tuber yield, tuber number per plant, and marketable rate. By contrast, the W3 treatment significantly reduces the marketable tuber percentage compared to W1 and W2, primarily due to a water deficit during critical growth stages, which increases the risks of secondary growth and tuber cracking (Deblonde and Ledent, 2001; Sweidan et al., 2023). Although the novel fertilizers enhance nutrient translocation to tubers through fulvic acid activity, their effectiveness is shown to be insufficient to counteract the negative impacts of severe water stress (W3) on yield components. These collective results indicate that water management has a more significant impact on yield formation parameters than fertilization.

### 4.4 Water and fertilizer use efficiency under different water-fertilizer regimes

Scientific water-fertilizer management practices have been demonstrated to significantly enhance the utilization efficiency of both water resources and fertilizers (Wang et al., 2020). Optimized irrigation strategies play a pivotal role in maximizing crop economic returns while ensuring efficient water allocation (Niu et al., 2024). Water use efficiency (WUE), which quantifies the crop’s capacity to convert irrigation water into biomass accumulation through optimized physiological processes, serves as a critical scientific basis for refining field irrigation management approaches (Shrestha et al., 2023). Kassaye et al. (2020) reported that water use efficiency (WUE) reaches its maximum under 50% deficit irrigation (DI), followed by 25% DI, with full irrigation showing the lowest WUE. This pattern aligns with the present findings where W2 treatment demonstrates the highest WUE under identical fertilization conditions, followed by W1, while W3 exhibits the lowest efficiency. This observation is further supported by Niu et al. (2024), who documented 10.0% and 31.6% significant improvements in WUE and irrigation water use efficiency (IWUE), respectively, under deficit irrigation compared to full irrigation in potato cultivation. Similar water-saving effects have been consistently validated across other crops including pepper (Bladia and AbdArahman, 2016)and eggplant (Ayas, 2017), indicating the broader applicability of controlled deficit irrigation strategies in improving water productivity. These findings further substantiate that moderate deficit irrigation can significantly enhance water use efficiency across multiple crop species without substantial yield reduction (Geerts and Raes, 2009; Shammout et al., 2018). Notably, under identical irrigation levels, the novel fertilizer treatments demonstrate significantly higher WUE and IWUE than control groups, suggesting that fulvic acid in the soil matrix improves plant water retention capacity through enhanced soil structure, root system development, and water-holding properties (Feng and Zhang, 2021; Man-Hong et al., 2020; Mosaad et al., 2024), thereby systematically improving overall water utilization efficiency. Drip fertigation demonstrates the capacity to enhance fertilizer partial factor productivity (PFP) while concurrently reducing water consumption. A nonlinear relationship between irrigation level and PFP is observed, with values initially increasing then decreasing as water application rises, reaching maximum efficiency (52.5-100.1 kg·kg^-1^) under the W2 treatment. These findings align with established research on water-nutrient interaction dynamics in crop production systems (Zhang et al., 2021). The W2F2 treatment achieves optimal partial factor productivity (PFP) in this study, demonstrating that ideal water-fertilizer management requires integrated optimization of both irrigation and fertilization parameters rather than single-factor evaluation. The enhanced PFP observed with fulvic acid-enriched novel fertilizers confirms their dual capacity to simultaneously reduce chemical fertilizer application rates while improving both nutrient use efficiency and crop yield performance.

### 4.5 Correlation and principal component analysis

Pearson correlation analysis of potato plant characteristics elucidates the effects of novel fertilizers under varying drip irrigation levels on potato growth and yield. Significant correlations are observed between plant height and multiple parameters including stem diameter (SD), aboveground dry weight (DW), relative chlorophyll content (SPAD), photosynthetic rate, yield components, and water-fertilizer use efficiency. These robust correlations demonstrate that fulvic acid-enriched water-soluble fertilizers substantially influence potato growth and productivity, potentially through activation of growth-promoting pathways under optimal irrigation, resulting in enhanced biomass accumulation, chlorophyll content, and yield-related traits. The consistent positive relationships among growth parameters, photosynthetic efficiency, resource use efficiency, and yield indicate multifaceted mechanisms of fulvic acid action, including improved photosynthesis, nutrient uptake, and water management, collectively contributing to yield enhancement. Principal component analysis (PCA), a well-established multivariate statistical method for evaluating parameter interrelationships, confirms W2F2 as the top-ranked treatment, exhibiting optimal performance in yield, WUE, and PFP. These comprehensive findings support the recommendation of W2F2 as the superior water-fertilizer management strategy for potato cultivation in southern Xinjiang.

## 5 Conclusion

This study demonstrates that both drip irrigation levels and novel fertilizer formulations have a significant impact on potato growth, yield, and water-fertilizer use efficiency. Water use efficiency (WUE) initially increases, then decreases with rising irrigation amounts, reaching peak efficiency under the W2F2 treatment. The application of novel fertilizers under identical irrigation regimes consistently enhances potato growth and yield. Compared to full irrigation (W1) and formula fertilizers F1 and F3, the moderate irrigation W2 combined with F2 formulation demonstrates superior performance in promoting plant development, improving both WUE and PFP, while simultaneously increasing yield by 121.29% and enhancing marketable tuber rate under 15% water-saving conditions. Comprehensive evaluation of growth parameters, yield components, and resource use efficiency identifies the top three management strategies as W2F2 > W2F3 > W1F1, with the optimal practice determined to be F2 formula fertilizer (fulvic acid: 60g·L^-1^, N-P-K: 130-120–150 kg·ha^-1^) applied at 3865.5 m^3^·ha^-1^ irrigation. It should be noted that in this experiment, the three newly formulated fertilizers (F1, F2, F3) differed in both fulvic acid (FA) concentration and NPK ratios. As a result, it was impossible to separate the effect of FA itself from the impact of changes in macronutrient content. Therefore, the effects observed in this study reflect the comprehensive impact of the new fertilizer formulations. Future research should adopt an is nutrient design—that is, treatments with or without FA addition based on identical NPK levels—to accurately clarify the specific role of FA in enhancing potato performance under deficit irrigation. Moreover, monitoring indicators should include changes in soil nitrogen, phosphorus, and potassium pools at different growth stages, combined with environmental indicators such as nitrogen leaching, to enable a more comprehensive evaluation of the benefits of this FA-containing water-soluble fertilizer.

## Data Availability

The original contributions presented in the study are included in the article/supplementary material. Further inquiries can be directed to the corresponding authors.
